# Use of the Healthy Lifestyle Coaching Chatbot App to Promote Stair-Climbing Habits Among Office Workers: Exploratory Randomized Controlled Trial

**DOI:** 10.2196/15085

**Published:** 2020-05-19

**Authors:** Meihua Piao, Hyeongju Ryu, Hyeongsuk Lee, Jeongeun Kim

**Affiliations:** 1 Office of Hospital Information Seoul National University Hospital Seoul Republic of Korea; 2 College of Nursing Seoul National University Jongno-gu Republic of Korea; 3 College of Nursing Gachon University Incheon Republic of Korea; 4 Interdisciplinary Program of Medical Informatics Seoul National University Seoul Republic of Korea; 5 Research Institute of Nursing Science Seoul National University Seoul Republic of Korea

**Keywords:** exercise, habits, reward, health behavior, healthy lifestyle

## Abstract

**Background:**

Lack of time for exercise is common among office workers given their busy lives. Because of occupational restrictions and difficulty in taking time off, it is necessary to suggest effective ways for workers to exercise regularly. Sustaining lifestyle habits that increase nonexercise activity in daily life can solve the issue of lack of exercise time. Healthy Lifestyle Coaching Chatbot is a messenger app based on the habit formation model that can be used as a tool to provide a health behavior intervention that emphasizes the importance of sustainability and involvement.

**Objective:**

This study aimed to assess the efficacy of the Healthy Lifestyle Coaching Chatbot intervention presented via a messenger app aimed at stair-climbing habit formation for office workers.

**Methods:**

From February 1, 2018, to April 30, 2018, a total of 106 people participated in the trial after online recruitment. Participants were randomly assigned to the intervention group (n=57) or the control group (n=49). The intervention group received cues and intrinsic and extrinsic rewards for the entire 12 weeks. However, the control group did not receive intrinsic rewards for the first 4 weeks and only received all rewards as in the intervention group from the fifth to twelfth week. The Self-Report Habit Index (SRHI) of participants was evaluated every week, and the level of physical activity was measured at the beginning and end of the trial. SPSS Statistics version 21 (IBM Corp) was used for statistical analysis.

**Results:**

After 4 weeks of intervention without providing the intrinsic rewards in the control group, the change in SRHI scores was 13.54 (SD 14.99) in the intervention group and 6.42 (SD 9.42) in the control group, indicating a significant difference between the groups (*P*=.04). When all rewards were given to both groups, from the fifth to twelfth week, the change in SRHI scores of the intervention and control groups was comparable at 12.08 (SD 10.87) and 15.88 (SD 13.29), respectively (*P*=.21). However, the level of physical activity showed a significant difference between the groups after 12 weeks of intervention (*P*=.045).

**Conclusions:**

This study provides evidence that intrinsic rewards are important to enhance the sustainability and effectiveness of an intervention. The Healthy Lifestyle Coaching Chatbot program can be a cost-effective method for healthy habit formation.

**Trial Registration:**

Clinical Research Information Service KCT0004009; https://tinyurl.com/w4oo7md

## Introduction

In a busy working life, lack of time for exercise is common among office workers. Given their occupational restrictions and difficulty in taking time off work, it is necessary to suggest effective ways for workers to exercise regularly. Nonexercise activities that can easily be performed in daily life have been introduced as an effective regimen. Nonexercise activity refers to the expending of energy via lifestyle physical activities, such as walking and stair climbing, which are naturally performed activities rather than intentional, planned, and structured activities [1]. Sustaining lifestyle habits that increase nonexercise activity in daily life can prevent weight gain even if effective weight loss cannot be expected. These physical activities are also simple behaviors that can be done habitually because they can be unconsciously repeated in daily life.

Until now, many models and theories have been proposed to explain healthy behavior in behavior-changing programs. However, these attempts are limited to cognitive areas and lack explanations for the sustainability of long-term solutions [2].

A habit formation model in which the habit is established by inducing repeated behaviors through cue-behavior-reward links in a consistent context has been suggested, and experiments are being conducted to support the effectiveness [3-5]. Habits require minimal deliberation or planning and can be enacted without conscious intention, and the key element for habit acquisition is context-specific repetition. This involves carrying out the target behavior repeatedly in the same situation to reinforce associations between the behavior and the situational cues [6]. Several treatment modalities have been designed and implemented using the habit formation model and have suggested a promising new tool to support measurable change in the way patients relate to food and physical activity. However, most of these have substantial barriers that undermine long-term strategies such as lack of adherence to the intervention, time constraints, and lack of consistent follow-ups over the long term [7].

The use of information and communication technologies, especially mobile apps, is demonstrating great potential in the delivery of treatment programs. These interventions are becoming highly valuable by promoting the continuous access of patients without the need for face-to-face meetings, home visits, or extra expenses [8]. However, most health-related apps have functions that provide information and tracking and record activity status, making continuous use difficult. The effectiveness tends to be low, with many users not using the app after downloading it and commonly deleting it after 1 month [9]. One of the reasons for this is lack of continuous motivation during management. Behaviors that require habit formation also require continuous motivation, and it is necessary to build an environment in which they form organic relationships with each other; thus, implementation of these functions in smartphone apps is necessary to provide an intervention.

In South Korea, the most popular messenger app, KakaoTalk, had 48.2 million active users as of the first quarter of 2015. KakaoTalk uses the smartphone network to deliver real-time communication in one-to-one or group chats and can be linked to services such as Kakao Plus Friend [10]. Although messenger apps are limited in functional aspects when compared with other apps, they supplement their limitations through habituation, convenience, interaction, social presence among network members, and active emotional exchange. They can therefore be used as a tool to provide a health behavior intervention that emphasizes the importance of sustainability and involvement. It is thus necessary to develop a mobile intervention delivery method that maintains interest and manages health behaviors from a long-term perspective. Thus, in this study, we aimed to assess the efficacy of the Healthy Lifestyle Coaching Chatbot intervention presented via a messenger app aimed at stair-climbing habit formation for office workers.

## Methods

### Study Design

A parallel study design was used for this exploratory trial that took place for 12 weeks from February to April 2018. Randomization was used to avoid contamination between the intervention and control groups. The research plan was reviewed and approved by the institutional review board of Seoul National University (IRB No. 1706/003-026), and the clinical trial was registered with the Clinical Research Information Service [KCT0004009].

### Recruitment

The sample size was calculated based on the power analysis formula [11]. With a significance level α=.05, power 1–β=0.80, and medium effect size=0.5 set based on a 1-tailed test, the minimum number needed for each group was 51. After assuming a 20% dropout rate, we calculated that each group needed 61 participants for a total of 122 research participants.

Participants were employees whose work was performed in an office or another administrative setting. We recruited those who understood the purpose of the research, wanted to participate voluntarily, and gave written consent. The inclusion criteria were those aged 24 years and older who could understand and respond to the survey content and had experience using the KakaoTalk app.

Participants were recruited from one of the advertising sharing platforms. With this platform, office workers could get information on companies, restaurants, events, and various community content. The recruitment details were uploaded on the platform event page. Once participants showed interest and agreed to join the study, they could connect to the KakaoTalk Plus Friend ID Healthy Lifestyle Coaching Chatbot through a QR code and click the “add friend” icon to be added as members automatically. An online consent form and survey link were then sent. Because only one KakaoTalk ID can be set per mobile phone number, it is possible to check the participants from manager account in real time.

During recruitment, 128 participants who read the research introduction signed up through QR code. After 7 who did not answer the questionnaire were excluded, 121 participants were randomized into 2 groups: 61 in the intervention group (IG) and 60 in the control group (CG). Block randomizations were performed using the R program (R Foundation for Statistical Computing). The number of subjects was set as 121, and the block size was set as 4.

**Figure 1:**
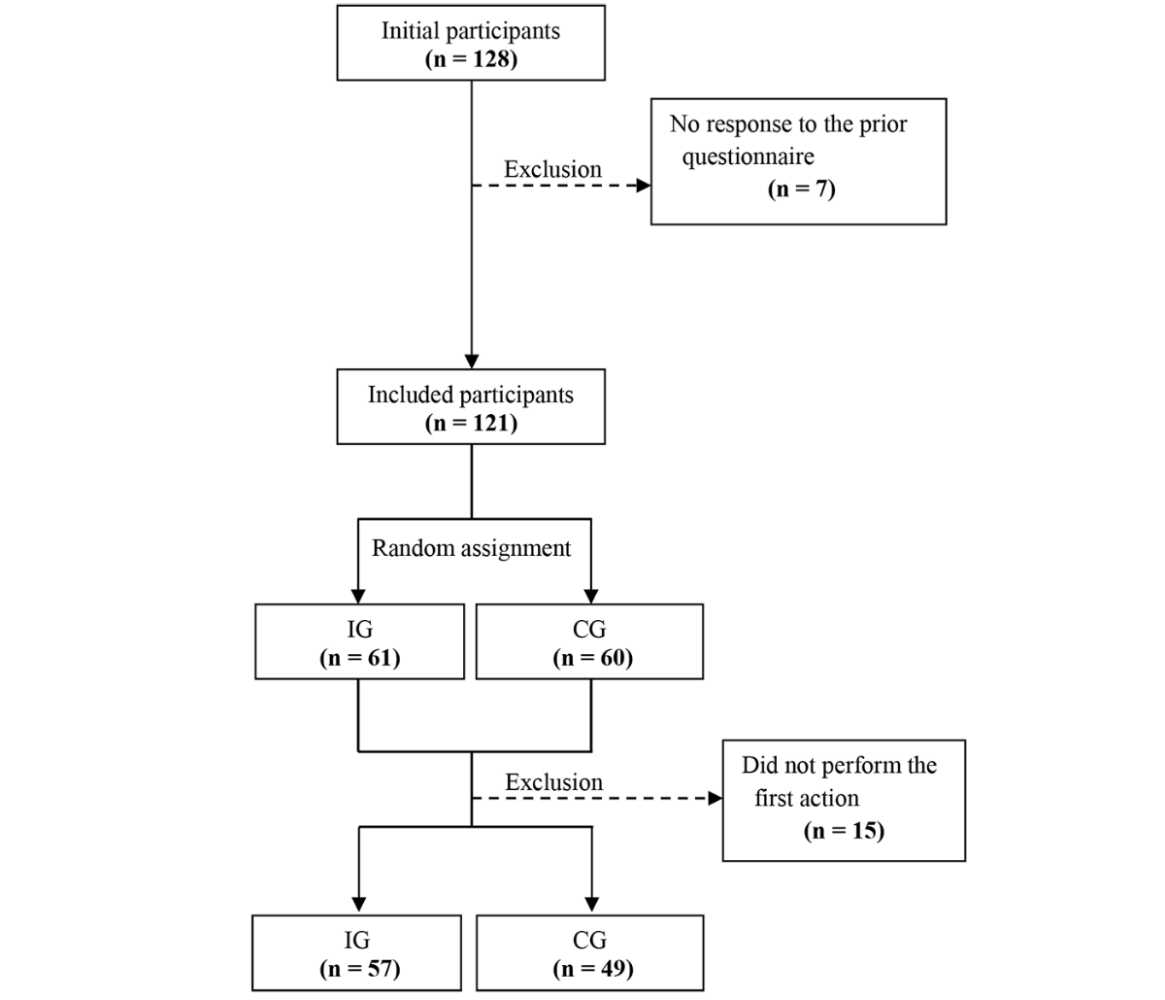
Flow of study participant enrollment.

Because performing the first action is important in habit formation, participants were asked to perform the stair-climbing and upload a proof shot. A total of 15 participants (4 in the IG and 11 in the CG) who did not upload a picture were considered nonexecuters of the first action and were excluded from the study. A total of 106 participants (57 in the IG and 49 in the CG) were included in the 12-week intervention program (Figure 1). The intervention was performed from February 1, 2018 to April 30, 2018.

### Messenger App-Based Intervention

The intervention Healthy Lifestyle Coaching Chatbot was presented via an app aimed at progressively establishing stair-climbing habits and increasing physical activity levels. The most popular messenger app in South Korea, KakaoTalk, was used as a platform for delivering the intervention. The functions were designed based on the habit formation model, including three elements: cue, behavior, and rewards (internal and external rewards). In addition, an automatically responding chatbot was developed using the Watson Conversation tool (IBM Corp), and it was linked to the KakaoTalk Smart Chat application programming interface (API) through the RESTful API.

In this study, cue was designed as a push alarm sent automatically to the participants every day to remind them to perform the stair-climbing behavior. Participants were asked to set realistic behavioral goals based on their daily routines, and their responses were designed as a push alarm. Through the KakaoTalk administrator messaging system, they can be individually set and automatically sent to a designated person at a specified time. The push alarms were composed of five basic conditions: who, when, where, what, and how much. For example, if a participant named John routinely arrives at the office at 9:00 am and wants to use the company building stairs to get to the fourth floor, the behavior plan should be set as who: John, when: 9:00 am, where: company building stairs, what: stair climbing, how much: fourth floor. The daily push alarm would then be sent at 8:50 am, 10 minutes before the planned time, and include the information above as a cue. Push alarms were designed to be sent from Monday through Friday, official work days. Rewards were considered reinforcers of cue-response associations. A fundamental distinction can be drawn between extrinsic rewards (eg, financial incentives) and intrinsic rewards (eg, pleasure, satisfaction) [12].

The chat scenario was designed and implemented through the Watson conversation launch tool to enable the automatic chat function. It was then linked to the app through API. Because it was linked to the Plus Friend Smart Chat API, participants could chat with the chatbot through the KakaoTalk app to receive feedback based on the behavior performed each day.

Based on the literature, we designed the scenario with two conditions. For extrinsic rewards, points and coffee coupons were provided. To encourage positive emotions, coffee coupons were sent when participants completed the first day goal-directed action. Also, a message was automatically sent at 8:00 pm every day. The participants interacted with the chatbot by chatting about the contents of their performance. If the planned behavior was completed, participants were provided with 50 points per day, and if they repeated this behavior more than 3 times a week, a coffee coupon was sent on Sunday.

**Figure 2:**
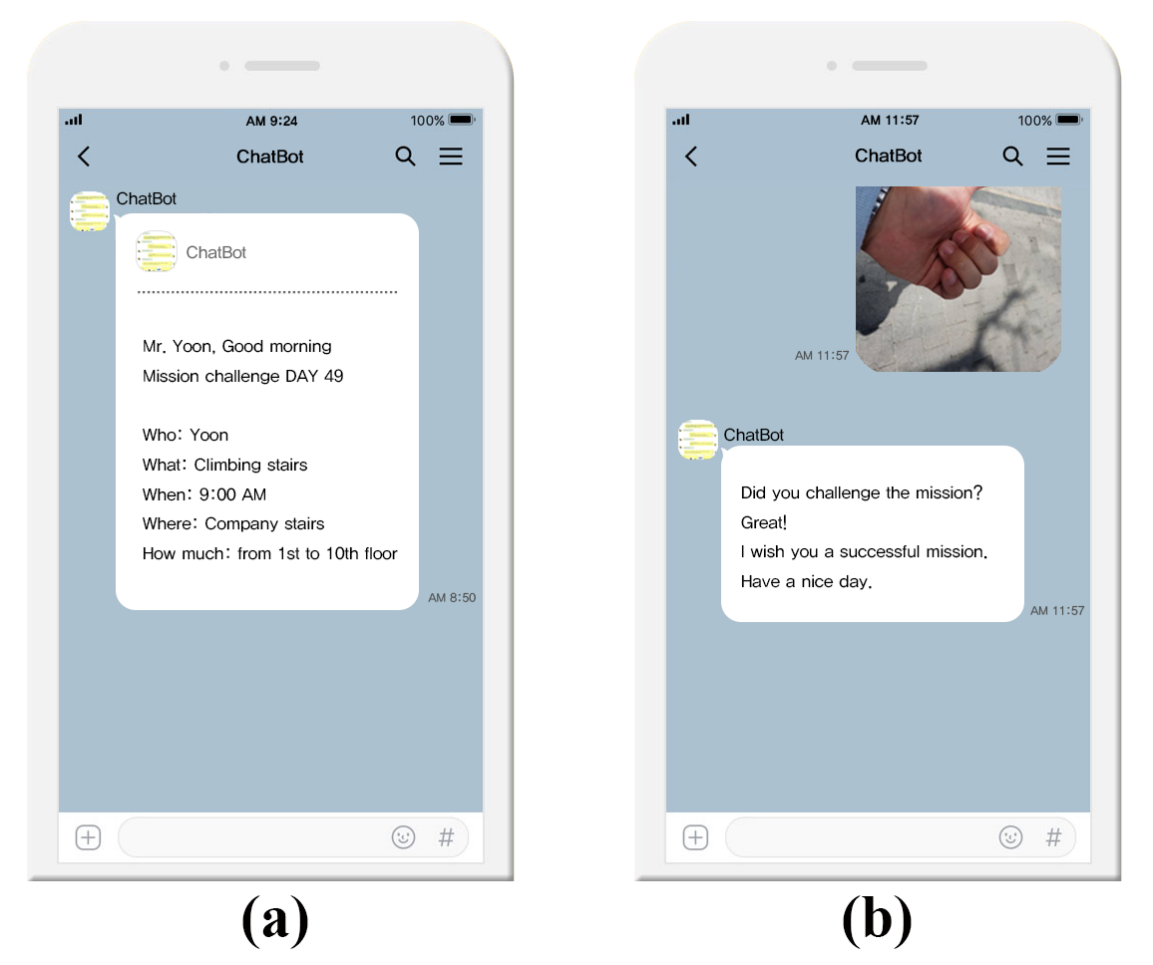
Screenshots of (a) push alarm reminders and (b) uploaded picture evidence and accomplishment with positive reinforcement as an intrinsic reward.

For intrinsic rewards, accomplishment and positive reinforcement were used. Participants were asked to take pictures once they finished the action and upload them to the chat room as evidence of the action, after which the chatbot would automatically send a compliment message providing positive feedback. Moreover, the pictures posted by other participants were collected, edited, and sent to participants every day to acknowledge that many participants joined together to challenge the mission and receive positive reinforcement. The intervention differed between the groups. Cues and intrinsic and extrinsic rewards were provided to the IG for the entire 12 weeks. However, intrinsic rewards were excluded for the first 4 weeks in the CG and added from the fifth to twelfth weeks (Figure 2).

### Outcome Measures

Habit is an automatic behavior gained via repetitive process. Because it was important to measure the habit strength of a particular action in our study, we measured the outcome using the Self-Report Habit Index (SRHI) [13]. SRHI is a tool that quantitatively measures the habit strength of a particular action and is composed of 12 items. Each item is measured on a 7-point Likert scale from 1=strongly disagree to 7=strongly agree, and the total score ranges from 7 to 84 points. Cronbach α reliability of this tool was .89 [14]. The survey for measuring SRHI was designed using Google Forms, and the link was sent to the participants in the messenger app every Sunday for 12 weeks. Participants could click the link and be taken to the survey pages.

### Statistical Analysis

SPSS Statistics version 21 (IBM Corp) was used for statistical analysis. The general characteristics of the participants were analyzed using frequency, percentage, mean, and standard deviation. Relevant statistical analyses were first performed to verify proper randomization (independent samples *t* test, chi-square test, and Fisher independent sample test). Repeated measures analysis of variance (ANOVA) with 12 moments was applied for measuring the changes in SRHI scores to evaluate the effect of the intervention on the two groups. Changes in physical activity levels between groups were tested with Fisher exact tests.

## Results

### Baseline Characteristics

Testing for homogeneity of general characteristics such as sex, age, physical activity status, weight control experience, weight, and hours sitting weekly between the IG and CG showed no significant differences in any of the baseline characteristics between the two groups (Table 1).

**Table 1:** Homogeneity test of general characteristics at baseline.

Characteristics		Intervention group (n=57)	Control group (n=49)	*t* test or chi-square	*P* value
**Sex, n (%)**				0.001	.92
	Male	25 (44)	21 (43)		
	Female	32 (56)	28 (57)		
**Age, n (%)**				5.06	.28
	20-29	9 (16)	2 (4)		
	30-39	26 (46)	29 (59)		
	40-49	16 (28)	12 (25)		
	50-59	6 (11)	5 (12)		
**Physical activity status, n (%)**				0.72	.70
	Low	22 (42)	20 (50)		
	Medium	28 (54)	18 (45)		
	High	2 (4)	2 (5)		
**Weight control experience, n (%)**				0.05	.82
	Yes	36 (63)	32 (65)		
	No	21 (37)	17 (35)		
Weight (kg), mean (SD)		64.15 (14.07)	64.47 (14.13)	–0.12	.91
Sitting hours weekly, mean (SD)		7.55 (2.21)	8.11 (2.58)	–1.17	.25

### Attrition Rate of Participants

In the IG, 2 participants out of 57 missed the follow-up due to overseas travel in the third week. In the CG, 11 participants out of 49 were considered to have dropped out of the study. Two missed the follow-up in the first week, 3 in the second week, 5 in the third week and 1 in the fourth week. Of them all, one reported the reason for terminating participation to be overseas travel, 1 reported hospitalization, and other 9 participants were automatically terminated due to consecutive weeks of nonresponse.

### Changes of Habit Strength

**Figure 3:**
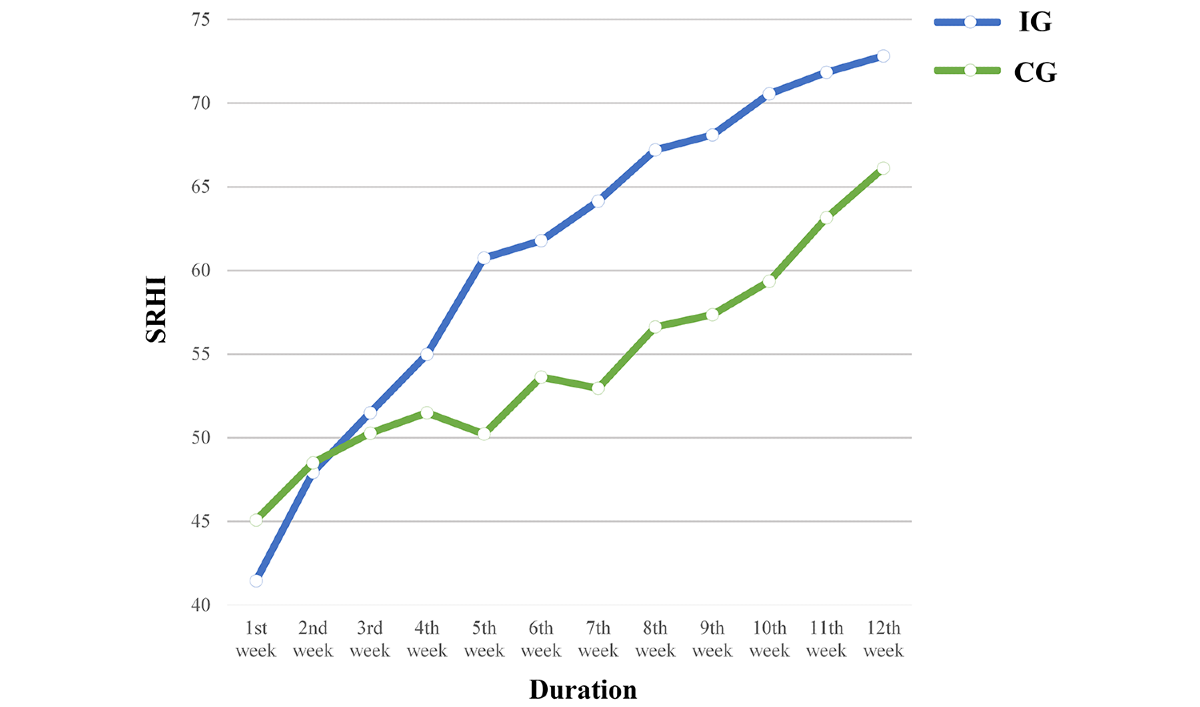
Plots of Self-Report Habit Index (SRHI) automaticity scores.

Over 12 weeks, the SRHI scores increased by an average of 31.38 points in the IG and 21.04 points in the CG on the 84-point scale (Table 2). Additionally, analysis of the change in SRHI scores for each group using repeated measures ANOVA showed statistically significant changes in the scores for both groups with an increase in the duration of the intervention (*P*<.001). In addition, the SRHI scores had significant between-group differences in the two groups (*P*=.008; Table 3 and Figure 3).

**Table 2:** Self-Report Habit Index characteristics based on group.

Intervention week	Intervention group, mean (SD)	Control group, mean (SD)
1	41.44 (15.69)	45.08 (14.39)
2	47.92 (16.40)	48.50 (13.66)
3	51.49 (16.47)	50.27 (13.66)
4	54..97 (16.92)	51.50 (13.60)
5	60.74 (14.62)	50.23 (11.96)
6	61.79 (14.53)	53.62 (13.33)
7	64.15 (14.37)	52.96 (16.26)
8	67.21 (14.80)	56.62 (15.37)
9	68.10 (11.64)	57.35 (10.39)
10	70.56 (10.06)	59.35 (10.39)
11	71.85 (9.71)	63.15 (10.68)
12	72.82 (9.77)	66.12 (7.15)

**Table 3:** Multivariate test results.

Effect		Value	*F* test	Hypothesis df^a^	Error df	Significance
**Habit**						
	Pillai’s trace	0.815	21.16	11	75	<.001
	Wilks’ lambda	0.185	21.16	11	75	<.001
Habit * Group						
	Pillai’s trace	0.359	2.70	11	75	.008
	Wilks’ lambda	0.641	2.70	11	75	.008

^a^df: degree of freedom.

### Effects of Intrinsic Rewards

After the fourth week of the intervention, the changes in the SRHI scores between the two groups were compared. The total change in SRHI scores had increased by 13.54 (SD 14.99) points in the IG and 6.42 (SD 9.42) points in the CG, and there were significant differences between the two groups during that period (*P*=.04).

After the fifth week, the intrinsic reward was added to the intervention in the CG, and the same intervention was provided to both groups from the fifth to twelfth week. We found that the total change in SRHI scores was 12.08 (SD 10.87) points in the IG and 15.88 (SD 13.29) points in the CG, with no significant difference in the changes between the two groups from the fifth to the twelfth week (*P*=.21; Table 4). This result indicated significant differences in SHRI score changes between the two groups for the first 4 weeks where no intrinsic reward was provided to the CG. However, all scores increased with no significant difference between the groups as a result of applying identical interventions to both groups by adding the intrinsic rewards to the CG from the fifth week. This indicated that the intrinsic reward acted as an influencing factor in habit formation.

**Table 4:** Self-Report Habit Index variation based on the difference of rewards.

Intervention duration	Variation	Intervention group, mean (SD)	Control group, mean (SD)	t(*p*)
Weeks 1 to 4	ΔSRHI^a^ (4–1)	13.54 (14.99)	6.42 (9.42)	2.12 (.04)
Weeks 5 to 12	ΔSRHI (12–5)	12.08 (10.87)	15.88 (13.29)	0.21 (.21)

^a^ΔSRHI: change in Self-Report Habit Index.

## Discussion

### Principal Findings

This study shows that the Healthy Lifestyle Coaching Chatbot program resulted in effective habit formation for stair-climbing behavior. Forming a habit is challenging, especially when transforming many things at once. In this study, the participants were asked to set small goals and take things one step at a time. Also, the behavior must be carried out repeatedly in the presence of the same contextual cues. Habit is an automatic behavior gained via repetition. In this study, most participants tended to increase the number of repetitions of the behavior and thus increase the habit strength as the intervention proceeded. This suggests that, according to the habit formation model hypothesis, given a consistent context, the repeated occurrence of cue-behavior-reward chains leads to increases in the strength of the habit behavior [15].

Based on the literature, a fundamental distinction can be drawn between extrinsic rewards (eg, financial incentives) and intrinsic rewards (eg, pleasure, satisfaction) [16]. The reward acts as motivation for sustaining a new behavior, so is an important factor in the repeated execution of behavior. However, previous research has suggested that providing simple financial rewards has limited success in sustaining motivational inducement. Although extrinsic rewards elicit motivation in the beginning, they eventually hinder a particular behavior from becoming habitual [16]. In this study, the SRHI score was 3.64 points higher in the CG after the first week oof the intervention. However, the upward trend until the fourth week was slower than the IG when only extrinsic reward was applied. Analysis of the SRHI score changes after the fourth week of the intervention showed that the changes were greater for the IG than for the CG at a statistically significant level. Furthermore, when similar rewards (including intrinsic and extrinsic) were provided to both groups from the fifth week on, the SRHI score showed a similar slope and increase without a significant between-group difference. This result concurs with other studies that found that extrinsic rewards elicit motivation regarding the behavior initially, but later the intrinsic rewards played an important factor for increasing the behavior continuation [17].

Lally et al [5] tracked the formation of healthy habits in a naturalistic setting, repeating a behavior in the presence of consistent cues. Based on the results, the habit formation tracked using SRHI was found to typically follow an asymptotic curve, and the initial repetitions caused large increases in SRHI scores, but with each new repetition, the score gains reduced until the behavior reached its limit of automaticity. Also, the average time for participants to reach the asymptote of automaticity was 65 days, which is near 10 weeks. In this study, changes in SRHI scores showed lower for the IG compared to the CG after week 5 even though there was no significant difference between the groups. We found that for the IG, the SRHI score increased by an average of 1 point after the tenth week. This result showed that it was possible for a repeated behavior to reach its maximum level of automaticity for the IG during the 12-week intervention. However, for the CG, the SRHI score increased by an average of 3 points after the tenth week. It is interesting to note that an intrinsic reward could help participants perform the behavior consistently enough to achieve habit status. The results of our study suggest the need for interventions using intrinsic reward factors to design an intervention program. Furthermore, positive feedback such as a compliment as an intrinsic reward is a simple but powerful tool in which the positive value of the reward can act as a motivational factor [16].

In this study, the messenger app KakaoTalk was used as an intervention delivery method. The KakaoTalk app uses the smartphone network to deliver real-time communication in one-to-one or group chats and can be linked to services such as chatbots. KakaoTalk has superior habituation, convenience, and interaction and is a platform in which social presence among network members and emotional exchanges are actively occurring [10]. With advancements in digital technology, mobile apps that help exercise management are being released, and wearable devices are becoming increasingly diverse. However, the dropout rate was considered to be high. One of the causes was continuous motivation that occurs during management.

In this study, 11 participants out of 49 in the CG and 2 participants out of 57 in the IG dropped out during the intervention; the total dropout rate was 12.26%. Also, all dropout cases appeared during the first 4 weeks. In comparison with previous studies in which dropout rates approached 50% at week 4, our study showed comparatively lower dropout rates. Although mobile interventions offer a promising way to deliver related content, dropout rates for this form may be high [18,19]. One potential contributing factor for this dropout rate is the difficulty in the use of the app, particularly when the app is designed to be used in a self-guided fashion [20].

This study designed the method of intervention delivery using a familiar app. Participants did not need to download other apps and learn how to use them. This delivery method is significant in that it minimized participant aversion, can provide sustained motivation, and can form an organic relationship.

For the duration of the intervention period, most participants dropped out in week 3 and week 4 during which the Lunar New Year holidays occurred. Based on prior research, lapses have an important influence on the sustainability of behavior. Although lapses of approximately 1 to 2 days do not greatly affect the sustainability of behavior, lapses of approximately 1 week greatly affect the sustainability of behavior and significantly lower the possibility of future execution of behavior [5]. We found that research subjects who lapsed for approximately 1 week due to the Lunar New Year on week 3 also showed high dropout rates in our study as well. This suggests the need to improve the effectiveness of the program by including intervention methods to provide motivation so that lapses do not exceed 1 week by specially managing the research subjects who are lapsing.

Thus, using a messenger app that allows a continuous relationship with the research subjects and provides motivation and professional advice is garnering much interest. Therefore, the methodology used in our study can contribute to enhancing the sustainability and effectiveness of care intervention by developing programs and delivery tools that implement theories on a wider variety of illnesses or health behaviors.

### Limitations

There were limitations in designing the program because the functionality provided by the current messenger app does not include direct communication functions, such as informing colleagues of their commitments and receiving positive support messages from others. Also, research should be done to implement different kinds of rewards to identify how the rewards affect the habits and which ones are the most important to maintain habits. Furthermore, there could be possible bias in the results since some studies have shown that participants who volunteer in these kinds of research are more motivated to be physically active or develop the habits.

### Conclusions

This study provides evidence that the Healthy Lifestyle Coaching Chatbot program presented via an app can be used to provide improvements in relevant variables in the long term and can be useful in producing new healthy habits. This messenger app as a delivery tool is considered a cost-effective means to deliver interventions to a large number of people.

## Appendix

Multimedia Appendix 1 CONSORT-eHEALTH checklist (V 1.6.1).

## Abbreviations

ANOVA: analysis of variance
API: application programming interface
CG: control group
IG: intervention group
SRHI: Self-Report Habit Index
